# Methylphenidate for Emotional Dysregulation in patients with Comorbid ADHD and Borderline Personality Disorder

**DOI:** 10.1192/j.eurpsy.2025.792

**Published:** 2025-08-26

**Authors:** S. Pardossi, A. Fagiolini, D. Koukouna, B. Firenzuoli, P. Carmellini, C. Pierini, M. Pinzi, V. Centi, F. Torrigiani, A. Cuomo

**Affiliations:** 1 Molecular Medicine, University of Siena, Siena, Italy

## Abstract

**Introduction:**

Emotional dysregulation (ED) is highly heterogeneous in its definition, presentation, and classification. It manifests in a variety of ways, including emotional impulsivity, heightened emotional intensity (both positive and negative), and emotional lability. This pattern of emotional experience and expression is seen in several psychiatric disorders, such as Attention-Deficit Hyperactivity Disorder (ADHD) and Borderline Personality Disorder (BPD). Methylphenidate (MTH) has been extensively studied for its efficacy in treating ED in patients with ADHD, but it has been less studied for specific dimensions of BPD, such as impulsivity and executive dysfunction, and even less studied for ED in patients with comorbid ADHD and BPD.

**Objectives:**

This retrospective observational study evaluates the efficacy of MTH in reducing ED in patients with a dual diagnosis of BPD and ADHD. Specifically, we report the efficacy of MTH on emotional impulsivity, emotional lability, and emotional intensity (both positive and negative).

**Methods:**

Patients with a dual diagnosis of ADHD and BPD treated with MTH were evaluated. Descriptive statistics were used to assess the presentation of the comorbidity. The Negative Emotion Dysregulation (NED) subscale of the Reactivity, Intensity, Polarity, and Stability Questionnaire (RIPoSt-40) was used to measure emotional lability and emotional intensity (both positive and negative). In addition, the C-T-Score representing ‘Impulsivity/Emotional Lability’ from the Conners’ Adult ADHD Rating Scales-Self Report: Short Version (CAARS-S:S), was assessed at baseline, week 2, and week 4. A repeated measures ANOVA test was used to assess mean differences.

**Results:**

Of the 24 patients (37.5% female, median age = 24, IQR: 21-37), 23 participants (96%) in the cohort had attention deficits, 9 participants (38%) had hyperactivity, and 19 participants (79%) had impulsivity. Most participants had overlapping symptom profiles (Figure 1). The median dose of MTH was 20 mg [10-30 mg], and the median duration of treatment was 4 months [3-10 months]. Statistically significant reductions in both NED and C-T-Score were observed from baseline to week 2 and from week 2 to week 4 (Table 1, Figure 2).

**Table 1:** 

	BASELINE mean (sd)	WEEK 2 mean (sd)	WEEK 4 mean (sd)	p-value
*NED*	122 (2.43)	105.92 (5.94)	91.83 (8.46)	0.0001
*C-T-Score*	74.54 (5.27)	66.92 (5.85)	62.88 (5.76)	0.0001

**Image 1:**

**Figure:**
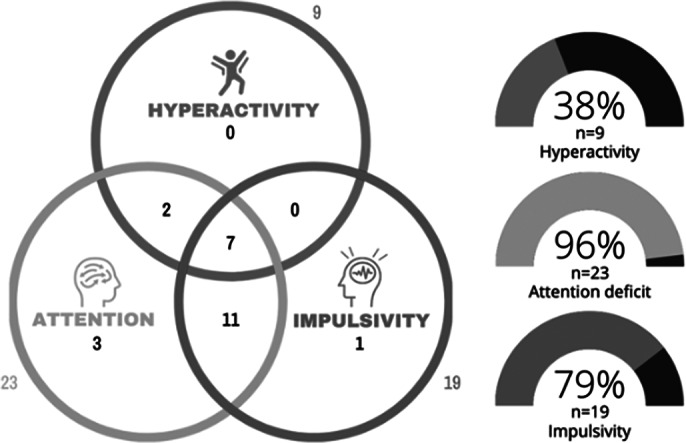

**Image 2:**

**Figure:**
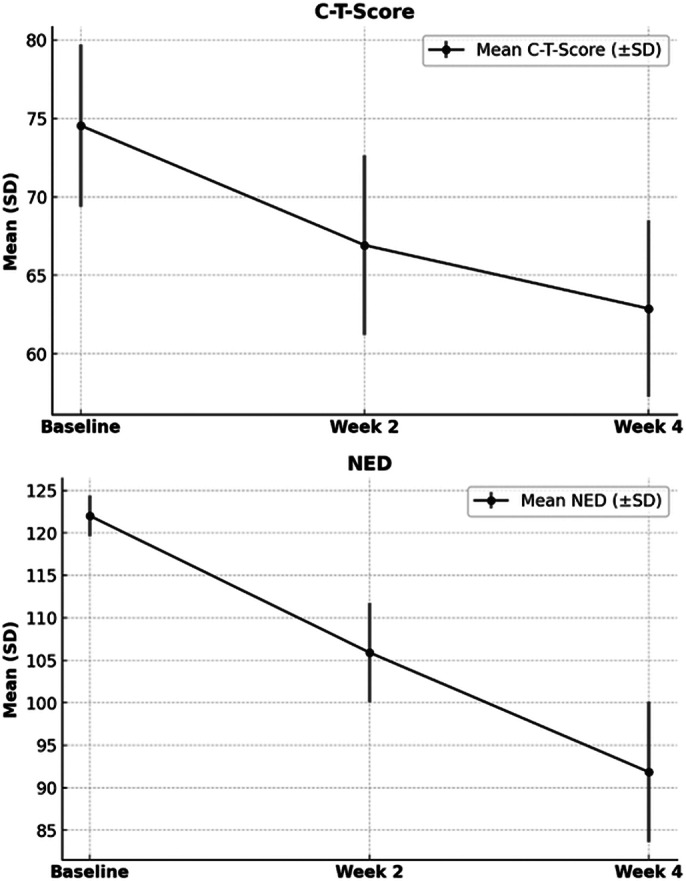

**Conclusions:**

ED is a core symptom of BPD and an important aspect of ADHD psychopathology. Targeting this pattern is complex because it encompasses multiple dimensions such as emotional impulsivity, lability, and intensity. In our study, MTH demonstrated efficacy in reducing these aspects in a real-world population with a dual diagnosis of ADHD and BPD, suggesting a possible role in improving ED, but further controlled studies are needed to confirm these findings.

**Disclosure of Interest:**

None Declared

